# From discovery to replication: Power and definitions matter for multiple sclerosis severity

**DOI:** 10.1177/13524585241265021

**Published:** 2024-07-26

**Authors:** Pernilla Stridh, Jesse Huang, Ingrid Kockum

**Affiliations:** Karolinska Neuroimmunology and Multiple Sclerosis Center, Department of Clinical Neuroscience, Karolinska Insitutet, Stockholm, Sweden

We appreciate the efforts by Campagna et al.^1^ to replicate the recently discovered first genetic variant associated with multiple sclerosis (MS) severity, rs10191329.^2^ They report a lack of association between rs10191329 and the median of three or more MS severity scores (MSSS) or age-related MS severity (ARMSS) scores in 1813 persons with MS (pwMS) from the MSBase cohort.

In our initial study, rs10191329 was associated with ARMSS after an average of 18 years of disease duration in 12,584 pwMS and independently replicated in 9805 pwMS, which remained significant in longitudinal analyses despite the modest effect size.^2^ This association was confirmed in another study, using ARMSS after a long disease duration (*p* = 0.035 in one-sided test),^3^ and has been associated with brain atrophy in pwMS.^4^

In an attempt to increase the power, Campagna et al. assigned the highest and lowest quintiles of median ARMSS and MSSS as binary severe and mild MS, respectively. Given the relatively small sample size and the modest effect size, we were surprised that their reported power was 81% for binary median ARMSS. Using the parameters from our replication cohort^2^ (Major Allele Frequency, MAF = 0.17, OR = 1.08), we estimated their power to be 17%. This stark difference underscores the need for well-powered replication studies with correctly estimated power to ensure accurate conclusions.

The authors claim that longitudinal data are more robust and an advantage of their design. Although we agree that serial assessments generally are preferential, the predictive power of assessments naturally increases with disease duration. This was the rationale for using Expanded Disability Status Scale (EDSS) in patients with long-standing disease.^2^ Serial assessments collapsed into a single median score, as done by Campagna et al., are analogous to the cross-sectional mid-point, which is not longitudinal and likely less informative than long-duration measures. We correlated the median MSSS and ARMSS to the last MSSS and ARMSS measure, as used in our study, in Swedish pwMS matching Campagna et al.’s inclusion criteria (*N* = 10,099) and found them to be highly correlated (ρ = 0.91, *p* < 2.2 × 10^−16^ for both), refuting the claim that the median of serial assessments captures severity significantly differently.

All severity measures (median MSSS/ARMSS, latest MSSS/ARMSS, and binned mild/severe disease) were significantly associated with rs10191329 with expected effect sizes in the Swedish cohort (*N* = 7172, models adjusted for age, sex, date of birth, genotyping array, and first 10 principal components). To confirm that the power was the source of difference between the two studies, we down-sampled our cohort to match Campagna et al.’s criteria. Like in their study, the associations were no longer significant, though effect estimates remained similar to those of the whole cohort (e.g. binary median ARMSS, *N* = 928, OR = 1.12, *p* = 0.4). This further illustrates that Campagna et al.’s lack of observed association with rs10191329 is most likely driven by insufficient power. Insufficient evidence is provided for their statement that the median of all scores is a more robust measure. Instead, we suggest that measures based on longer disease duration are more advantageous, likely because they give more time for genotype differences to manifest.
